# Carrier density effect on recombination in PTB7-based solar cell

**DOI:** 10.1038/srep13648

**Published:** 2015-09-01

**Authors:** Yutaka Moritomo, Kouhei Yonezawa, Takeshi Yasuda

**Affiliations:** 1 Graduate School of Pure and Applied Science, Univ. of Tsukuba, Tsukuba 305-8571, Japan; 2 Center for Integrated Research in Fundamental Science and Engineering (CiRfSE), Univ. of Tsukuba, Tsukuba 305-8571, Japan; 3 Photovoltaic Materials Unit, National Institute for Materials Science (NIMS), Tsukuba, Ibaraki 305-0047, Japan

## Abstract

Organic solar cells (OSCs) are promising alternatives to the conventional inorganic solar cells due to their low-cost processing and compatibility with flexible substrates. The development of low band-gap polymer, *e.g*., poly-[[4,8-bis[(2-ethylhexyl)oxy]benzo[1,2–b:4,5–b’]dithiophene-2,6-diyl] [3-fluoro-2-[(2-ethylhexyl)carbonyl]thieno[3]thiophenediyl]] (PTB7), increases the power conversion efficiency (PCE) in the last decade. Here, we investigated the interrelation between the instantaneous carrier density (*n*) per donor (D)/acceptor (A) interface area and the carrier density (*n*_collected_) collected as photocurrent in PTB7/C_70_ heterojunction (HJ) device. By means of the time-resolved spectroscopy, we confirmed that the exciton—to—carrier conversion process takes place within ~1 ps at the D/A interface of the PTB7/C_70_ HJ device. We further determined the absolute magnitude of *n* by combination of the time-resolved and electrochemical spectroscopies. We found that the carrier recombination becomes dominant if *n* exceeds a critical concentration (*n*_c_ = 0.003 carriers/nm^−2^). We confirmed that a similar behaviors is observed in the PTB7/[6,6]-phenyl C_71_-butyric acid methyl ester (PC_71_BM) bulk heterojunction (BHJ) device. Our quantitative investigation based on the HJ device demonstrates that the fast carrier escape from the D/A interface region is indispensable for high PCE, because the carrier accumulation nonlinearly accelerates the carrier recombination process.

In OSCs, the carriers are produced by the exciton dissociation at the D/A interface in the active layer, reflecting the weak screening effect and/or strong exciton effect (Frenkel exciton)^1^. In other words, the carriers are localized at the interface at the initial stage of the photovoltaic process. This makes a sharp contrast with the conventional inorganic solar cells (ISCs): The carriers are produced at every positions of the active layer, reflecting the strong screening effect and/or weak exciton effect (Wannier exciton). Then, the quantitative determination of the branching ratio between the carrier recombination at the interface region and the carrier escape from the region is indispensable for true comprehension of the photovoltaic process of OSCs. The escaped carriers will be collected at the collector electrodes. We propose that we can quantitatively investigate the carrier recombination/escape branching ratio by preparing the instantaneous dense carrier states by photo-excitation with femtosecond light pulses. Especially, in the HJ device, the instantaneous carrier density (*n*) per D/A interface area can be determined because the interface area is the same as the device area.

BHJ-type OSCs^2^,^3^, in which nano-level mixture of electron-donating polymer and electron-accepting fullerene-derivative provides a wide exciton-dissociative region within the active layer, show high PCEs and are promising alternatives to the conventional ISCs. Especially, the development of low band-gap polymer, *e.g*., PTB7, increases the PCE up to 9%^4^,^5^,^6^ in the last decade^7^,^8^. The increase in PCE stimulates extensive time-resolved spectroscopic investigations of the low-band gap OSCs to reveal the carrier formation process^9^,^10^,^11^,^12^. The femtosecond time-resolved spectroscopy is a powerful tool to reveal the carrier formation process, because the spectroscopy monitors the relative numbers of the photo-created excitons and carriers in the time domain. Significantly, the spectroscopy decouples the carrier formation and transfer processes, because the former process completes within several ps. Actually, the exciton-to-carrier conversion process in PTB7/PC_71_BM blend film completes in 0.2–0.5 ps^9^,^10^. Recently, a spectroscopic method^13^ was proposed to evaluate the carrier formation efficiency (Φ_CF_). Φ_CF_ is defined by *n*_formed_/*n*_photon_, where *n*_formed_ (*n*_photon_) is the density of the instantaneously formed carriers (absorbed photons) per unit area of the device. *n*_formed_ includes the weakly-bound state of the carriers. Absolute magnitude of *n*_formed_ can be estimated by the combination of the time-resolved and electrochemical spectroscopies. Φ_CF_ is the same as the exciton quenching efficiency, if all the quenched excitons are converted to carriers.

In this paper, we investigated the branching ratio between the carrier recombination at the interface region and the carrier escape from the region in the PTB7/C_70_ HJ device. By means of the time-resolved spectroscopy, we confirmed that the exciton-to-carrier conversion process takes place within ~1 ps at the D/A interface in the PTB7/C_70_ HJ device. In the PTB7/C_70_ HJ device, Φ_CF_ (=0.56) is nearly constant against the excitation pulse energy (*I*_photon_) per unit area of the device while the internal quantum efficiency (Φ_IQ_) steeply decreases with increase in *I*_photon_ beyond 0.8 μJ/cm^2^. We further determined the interrelation between the instantaneous carrier density (*n*) per D/A interface area and the carrier density (*n*_collected_) collected as photocurrent. We found that the carrier recombination becomes dominant if *n* exceeds a critical concentration (*n*_c_ = 0.003 carriers/nm^2^). A phenomenological rate equation with the carrier recombination term (−α*n*^3^) well reproduces the experimental relation between *n* and *n*_collected_. We confirmed that a similar behavior is observed in the PTB7/PC_71_BM BHJ device even though *n*_c_ is considered to be much lower than the value of the HJ device.

## Results

### Pulse energy dependence of Φ_IQ_ of PTB7-based OSCs

We fabricated the two types of the PTB7-based OSCs: PTB7/C_70_ HJ solar cell with a structure of indium tin oxide (ITO)/poly-(3,4-ethylenedioxythiophene) (PEDOT): poly-(styrenesulfonate) (PSS) (40 nm)/PTB7 (18 nm)/C_70_ (25 nm)/bathocuproine (BCP) (5 nm)/MgAg and PTB7/PC_71_BM BHJ cell with a structure of ITO/PEDOT:PSS (40 nm)/active layer (89 nm)/LiF (1 nm)/Al. We measured current (*J*)—voltage (*V*) curves (see Supplementary Fig. S1 online) of the two OSCs. The HJ device exhibits an open circuit voltage (*V*_oc_) of 0.68 V, a short circuit current (*J*_sc_) of 5.9 mA/cm^2^, a fill factor (FF) of 0.68, and a PCE of 2.7%, while BHJ device exhibits a *V*_oc_ of 0.74 V, a *J*_sc_ of 17.7 mA/cm^2^, a FF of 0.65, and a PCE of 8.5%. The FF value (=0.68) of the HJ device is slightly higher than that (=0.65) of the BHJ device. The suppressed FF value of the BHJ device is ascribed to the efficient carrier recombination during the carrier transfer process, reflecting the nano-level mixing of the D and A domains. We measured incident photon-to-current conversion efficiency (IPCE) spectra (see Supplementary Fig. S2 oneline) of the two OSCs. The IPCE of the HJ device is nearly the half of that of the BHJ device. The suppression is ascribed to the thinner active layer (43 nm) of the HJ device as compared with that (89 nm) of the BHJ device. The thinner active layer is further responsible for the lower PCE and *J*_sc_ values of the HJ device.

**Figure 1:**
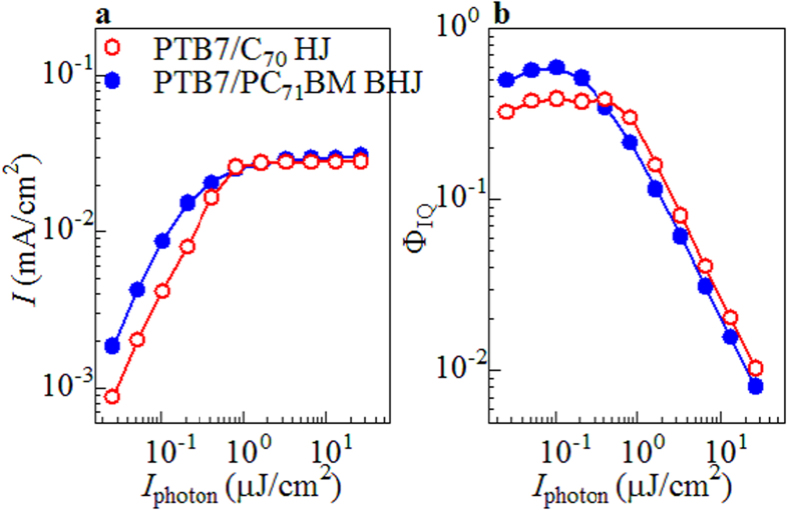
(**a**) Photocurrent (*I*) against energy (*I*_photon_) of femtosecond light pulse at 400 nm in the PTB7/C_70_ HJ and PTB7/PC_71_BM BHJ devices. (**b**) Φ_IQ_ against *I*_photon_. The magnitudes of Φ_IQ_ were estimated with considering the absorption indexes: 0.61 and 0.84 for HJ and BHJ devices, respectively. The curves are merely the guides to the eyes.

Figure 1a shows *I* against *I*_photon_ of femtosecond light pulse at 400 nm in the PTB7-based HJ and BHJ devices. In both the devices, the magnitude of *I* linearly increases with *I*_photon_ in the low-*I*_photon_ region below ~0.2 μJ/cm^2^. However, *I* becomes nearly constant (~0.03 mA/cm^2^) above ~2 μJ/cm^2^. A similar saturation of *I* against *I*_photon_ is observed in P3HT/PCBM BHJ device^14^. Figure 1b shows Φ_IQ_ at 400 nm against *I*_photon_. The magnitudes of Φ_IQ_ were evaluated from *I* and *I*_photon_ with considering the absorption indexes: 0.61 and 0.84 for HJ and BHJ devices, respectively. In the HJ device (open circles), the Φ_IQ_ value (~0.4) in the low- *I*_photon_ region is nearly the same as the value (=0.59) determined under 400 nm monochromatic radiation of 6.6 mW/cm^2^. The Φ_IQ_ value exponentially decreases with increase in *I*_photon_ beyond 0.8 μJ/cm^2^. In the BHJ device (closed circles), the Φ_IQ_ value (~0.6) in the low- *I*_photon_ region is close to the value (=0.79) determined under 400 nm monochromatic radiation of 6.6 mW/cm^2^. The Φ_IQ_ value exponentially decreases with increase in *I*_photon_ beyond 0.2 μJ/cm^2^.

### Carrier formation dynamics in PTB7/C_70_ heterojunction

**Figure 2:**
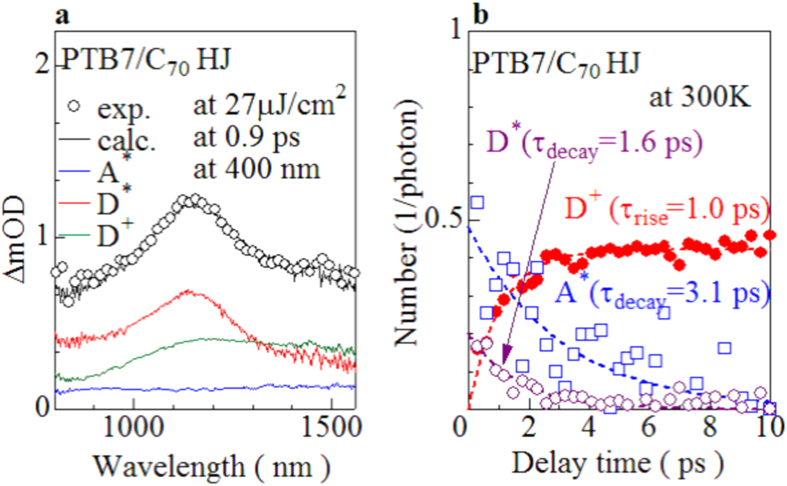
(**a**) ΔOD spectra (open circles) of PTB7/C_70_ bilayer film at 0.9 ps and at 400 nm together with the spectral decomposition into acceptor exciton (A^*^: blue curve), donor exciton (D^*^: green curve), and donor carrier (D^+^ : red curve) components. The ΔOD spectra of the PC_71_BM neat, PTB7 neat, and PTB7/C_70_ bilayer films at 1, 1, and 10 ps are regarded as the A^*^, D^*^, and D^+^ components, respectively. (**b**) Absolute number of acceptor exciton (*n*_A*_), donor exciton (*n*_D*_) and donor carrier (*n*_D+_) per an absorbed photon against the delay time. The magnitudes of *n*_A*_, *n*_D*_ and *n*_D+_ were evaluated by the spectral decomposition of the ΔOD spectra of the PTB7/C_70_ bilayer film. The solid curves are results of the least-squares fittings with an exponential function.

We investigated ΔOD spectra of PTB7/C_70_ bilayer, PC_71_BM neat, and PTB7 neat films at 400 nm (see Supplementary Fig. S3 online). The ΔOD spectra of the PC_71_BM (PTB7) neat film show a characteristic photoinduced absorption (PIA), which should be ascribed to the acceptor exciton (A^*^) [donor exciton (D^*^)] . The ΔOD spectra of the PTB7/C_70_ bilayer film show a broad PIA centered at 1150 nm. In the late stage (>10 ps), the profile of the PIA is essentially unchanged. In addition, the spectral profile is similar to that of the doping-induced spectrum of the PTB7 neat film (see Supplementary Fig. S4 online). Therefore, we ascribed the PIA to the photo-created donor carriers (D^+^). In the early state (<10 ps), however, an additional absorption component is observed around 1500 nm. The additional component should be ascribed to the PIAs due to A^*^ and D^*^. We decomposed the ΔOD spectra (ϕ_exp_) of the PTB7/C_70_ bilayer film into the components of A^*^ (ϕ_A*_), D^*^ (ϕ_D*_), and D^+^ (ϕ_D+_). The ΔOD spectra of the PC_71_BM (PTB7) neat film at 1 ps was regarded as ϕ_A*_ (ϕ_D*_) while the ΔOD spectra of the PTB7/C_70_ bilayer film at 10 ps was regarded as ϕ_D+_. The spectral weights of the respective components were evaluated by least-squares fitting of the observed spectra (ϕ_exp_) with the linear combination of ϕ_A*_, ϕ_D*_, and ϕ_D+_ : ϕ_cal_ = *C*_A*_ϕ_A*_ + *C*_D*_ϕ_D*_ + *C*_D+_ϕ_D+_. The coefficients, *C*_A*_, *C*_D*_ and *C*_D+_, were determined so that the evaluation function, 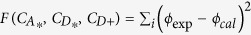, becomes the minimum. Figure 2a show an example of the spectral decomposition at 0.9 ps. We clearly observed that the photo-excitation at 400 nm excites D^*^ in addition to A^*^.

Now, let us evaluate the absolute numbers of the acceptor exciton (*n*_A*_), donor exciton (*n*_D*_) and donor carrier (*n*_D+_) per absorbed photon from the ΔOD spectra of the PTB7/C_70_ bilayer film. For this purpose, we need the spectral intensities, α_A*_, α_D*_, and α_D+_, per unit densities of A^*^, D^*^, and D^+^. In order to determine α_A*_ and α_D*_, we assumed that one absorbed photon creates one A^*^ (D^*^) in the PC_71_BM (PTB7) neat film. We determined α_A*_ (α_D*_) with use of the ΔOD spectra of PC_71_BM (PTB7) neat film (see Supplementary Fig. S3 online): α_A*_ (α_D*_) is evaluated to be 0.002 nm^2^/photons (0.020 nm^2^/photons) with considering absorption indexes. Then, *n*_A*_ (*n*_D*_) is calculated by (*I*_A*_/*n*_photon_)/α_A*_ [(*I*_D*_/*n*_photon_)/α_D*_], where *I*_A*_ (*I*_D*_) and *n*_photon_ are the intensity of the (A^*^) D^*^ component in the PTB7/C_70_ bilayer film and the absorbed photon number per unit density of the device. The magnitude of α_D+_ was determined from the electrochemical differential (ΔOD_EC_) spectrum (see Supplementary Fig. S4 online). α_D+_ is evaluated to be 0.013 nm^2^/carriers with use of the doped carrier density. Then, *n*_D+_ is calculated by (*I*_D+_/*n*_photon_)/α_D+_, where *I*_D+_ is the intensity of the D^+^ component in the PTB7/C_70_ bilayer film. The magnitudes of *I*_A*_, *I*_D*_, and *I*_D+_ were evaluated by the spectral decomposition of the ΔOD spectra into the A^*^, D^*^, and D^+^ components, as exemplified in Fig. 2a.

Figure 2b shows the absolute numbers of A^*^ (*n*_A*_), D^*^ (*n*_D*_) and D^+^ (*n*_D+_) per an absorbed photon against the delay time. The *n*_A*_ values significantly scatter reflecting the small cross section of A^*^ (α_A*_ = 0.002 nm^2^/photons). Nevertheless, we observed steep decreases in *n*_A*_ and *n*_D*_ with time. The decay times (τ_decay_) of *n*_A*_ and *n*_D*_ are roughly evaluated to be 3.1 and 1.6 ps, respectively. On the other hand, *n*_D+_ exponentially increases with the rise time (τ_rise_) of 1.0 ps. The rather slow decay time (τ_decay_ = 3.1 ps) of A^*^ suggests that the late decay component (>1 ps) of A^*^ does not contribute to the carrier formation. Actually, the sum of the initial exciton number, *i.e.*, *n*_A*_ + *n*_D*_ (~0.7/photon) is slightly larger than the carrier number (*n*_D+_ ~ 0.5/photon). Thus, the exciton-to-carrier conversion process in the PTB7/C_70_ bilayer film takes place at the D/A interface within ~1 ps. The carrier formation time (=~1 ps) in the PTB7/C_70_ bilayer film is longer than that (=0.2–0.5 ps) of PTB7/PC_71_BM blend film^10^. The longer conversion time is ascribed to be the longer exciton diffusion to the D/A interface in the HJ device than that in the BHJ device.

### Pulse energy dependence of Φ_CF_ in PTB7-based OSCs

**Figure 3:**
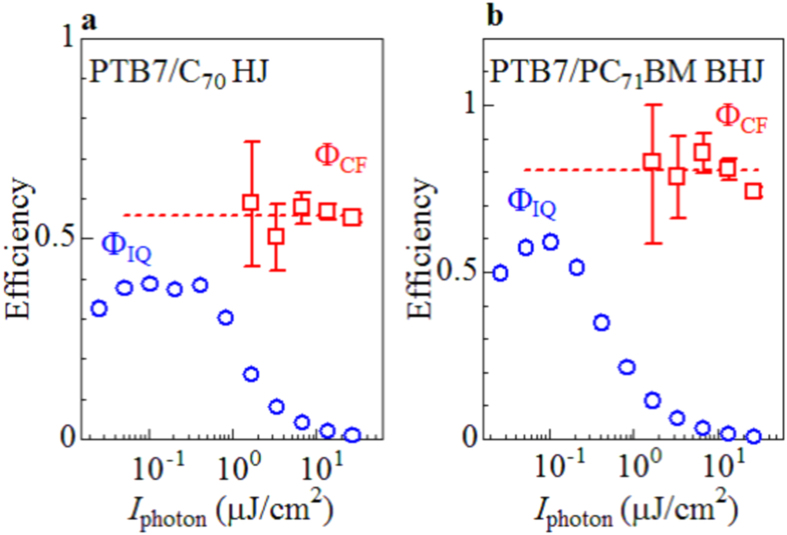
Φ_CF_ and Φ_IQ_ of (a) PTB7/C_70_ HJ and (b) PTB7/PC_71_BM BHJ devices against energy (*I*_photon_) of femtosecond light pulse at 400 nm. The magnitudes of Φ_CF_ were estimated with considering the absorption indexes: 0.37 and 0.60 for the bilayer and blend films, respectively. The error bars of Φ_CF_ were roughly evaluated from the signal/noise ratio of the femtosecond time-resolved spectra. The magnitudes of Φ_IQ_ were estimated with considering the absorption indexes: 0.61 and 0.84 for HJ and BHJ devices, respectively. The broken lines are merely the guide to the eyes.

Next, we investigated pulse energy dependence of the ΔOD spectra at 10 ps of the PTB7/C_70_ bilayer and PTB7/PC_71_BM blend films at 400 nm (see Supplementary Fig. S5 online). The magnitude of Φ_CF_ (=α_photon_/α_D+_) can be calculated from the two quantities. α_photon_ (=*I*_D+_/*n*_photon_) is the spectral intensities due to D^+^ per unit density of absorbed photons. α_D+_ is reported to be 0.013 nm^2^/carriers for PTB7^13^. In the PTB7-based OSCs, the carrier formation time is less than 1 ps. Therefore, we evaluated the α_D+_ values from the ΔOD spectra at 10 ps of the PTB7/C_70_ bilayer and PTB7/PC_71_BM blend films with considering the absorption indexes: 0.37 and 0.60 for the bilayer and blend films, respectively. Thus obtained Φ_CF_ values are plotted in Fig. 3 together with Φ_IQ_ against *I*_photon_. We found that Φ_CF_ (=0.56) is nearly independent of *I*_photon_ in the PTB7/C_70_ bilayer film even though Φ_CF_ at *I*_photon_ < 2 μJ/cm^2^ is difficult to determine. This makes a sharp contrast with a steep decrease in Φ_IQ_ with increase in *I*_photon_ beyond 0.8 μW/cm^2^. A similar behavior of Φ_IQ_ and Φ_CF_ is observed in the PTB7/PC_71_BM BHJ device. In the high-*I*_photon_ region, most of the carriers recombine and do not contribute to the photocurrent (Φ_IQ_ ≪ 1). We note that the carrier recombination process takes several nanoseconds in both the devices (see Supplementary Fig. S6 online). We tentatively evaluate the decay times (τ_decay_) by least-squares fittings with exponential functions, *i.e*., ΔmOD = *A*exp(−*t*/τ): τ_decay_ = 3.4 and 5.8 ns for the HJ and BHJ devices, respectively.

Here, we define the instantaneous carrier density (*n*) per unit area of the D/A interface. In the HJ device, the magnitude of *n* is the same as *n*_D+_ (=*n*_photon_ × Φ_CF_) because the D/A interface area is the same as the device area. In the BHJ device, however, the magnitude of *n* is smaller than *n*_D+_ because the interface area is much wider than the device area. Under the femtosecond pulse excitation, the carriers are instantaneously produced at the D/A interface before they start migration toward the collector electrodes. Actually, the carriers are generated at the D/A interface within ~1 ps. For example, the local carrier density (*n*) at the interface of the HJ device is ~0.1 carriers/nm^2^ at 10 μJ/cm^2^. Under a conventional continuous wave (CW) excitation, however, the carrier migration effectively reduces the *n* value. For example, the local carrier density at the interface is ~10^−2^ carriers/nm^2^ at 100 mW/cm^2^ if the carrier escape time from the interface region is 1 ns. On the other hand, it becomes ~1 carriers/nm^2^ at 100 mW/cm^2^ with the carrier lifetime of ~1 ms, which is comparable to the value (~0.1 carriers/nm^2^) under the femtosecond pulse excitation at 10 μJ/cm^2^. These arguments indicate that the significant carrier recombination efficiency observed in the high-*I*_photon_ region should be ascribed to the local dense carriers at the interface. In other words, the carriers at the interface region are amenable to the recombination while the carriers escaped from the region are free from the recombination.

## Discussion

**Figure 4:**
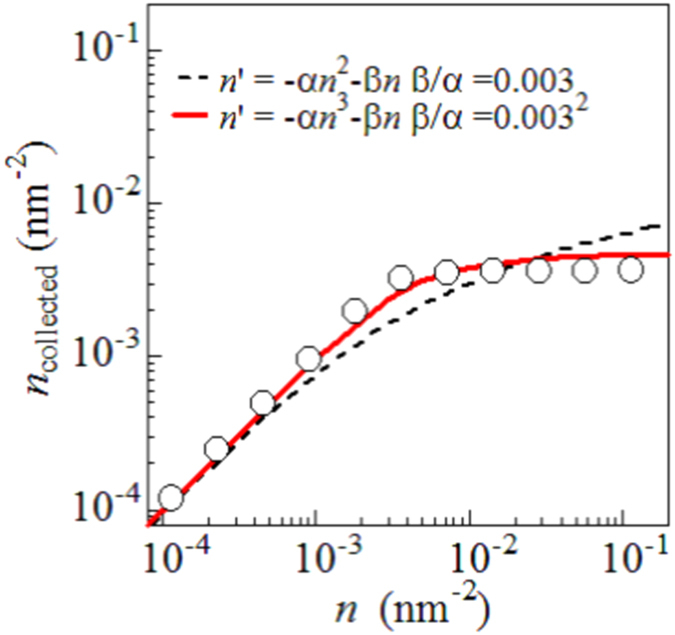
Carrier density (*n*_collected_) collected as photocurrent against instantaneous carrier density (*n*) per unit area of the D/A interface in PTB7/C_70_ HJ device. The *n* values were evaluated with assuming constant Φ_CF_ (=0.56). The solid and broken curves are drown by a phenomenological model (see text).

Figure 4 shows the carrier density (*n*_collected_) collected as photocurrent against *n* (=Φ_CF_ × *n*_photon_) in the HJ device. The *n* values were evaluated with assuming constant Φ_CF_ (=0.56). In the dilute-*n* region, the photocurrent is proportional to *n*. In the dense-*n* region, however, the photocurrent becomes nearly constant. This suggests that there exists a critical carrier density (*n*_c_ = 0.003 carriers/nm^2^) above which the carrier recombination becomes dominant. The *n*_c_ value (=0.003 carriers/nm^2^) corresponds to the value, in which one carrier exists every 18 × 18 nm^2^ squares. To quantitatively analyze the carrier recombination kinetics against *n*, we adopted a phenomenological rate equation: d*n*/dt = −α*n*^N^ − β*n*. The first and second terms represent the carrier recombination and the carrier escape from the interface region. The first term at N = 2 describes the conventional electron—hole recombination process. The ratio between the coefficients, α and β, should be fixed as β/α = *n*_c_^N−1^, because the first and the second terms become comparable at *n* = *n*_c_. We assume that all the escaped carriers contribute the photocurrent, *i.e.*, *n*_collected_ = ∫β*n*d*t*. The solid and broken curves in Fig. 4 are results of the phenomenological model at *N* = 3 and 2, respectively. We found that the curve at *N* = 3 excellently reproduces the experiment data. The deviation from the conventional *n*^2^-dependence (*N* = 2) is probably ascribed to the electric double layer effect at the interface, which may cause a many-body interaction between the carriers. Here, we note that the recombination/escape branching ratio is determined by the ratio not by the magnitudes of the coefficients, α and β. The magnitude determines only the time scale of the branching kinetics. That is, we have no adjustable parameter in this phenomenological mode.

A similar relation between *n* and *n*_collected_ is observed in the PTB7/PC_71_BM BHJ device even though we cannot evaluate the magnitude of *n*. Alternatively, we estimated the critical carrier density (*n*_c_’ = 0.002 carriers/nm^2^) per unit area of the device, above which the carrier recombination becomes dominant. We note that *n*_c_ (per unit area of the interface) is much smaller than *n*_c_’ (per unit area of the device) because the D/A interface area is much wider than that of the device area. That is, *n*_c_ of the BHJ device is much lower than *n*_c_ (=0.003 carriers/nm^2^) of the HJ device. We ascribe the suppressed *n*_c_ of the BHJ device to the unevenness of the D/A interface due to the to the nano-level mixing of the D and A domains^15^,^16^. Hedley *et al.*^15^ reported that the domain (100–200 nm) of PTB7/PC_71_BM blend film consists of small fullerene spheres (20–60 nm) inside the domain. The resultant inhomogeneity of the interface activates only a portion of the interface to dissociate the excitons. Then, the interface inhomogeneity effectively reduces the interface area and increases *n* (per unit area of the interface). In addition, the inhomogeneity causes the carrier trapping at the interface region. The carrier trapping should enhance the carrier recombination probability. On the other hand, the interface of HJ device, which is prepared by vacuum evaporation process, is considered to be much smoother. Actually, the root-mean-square (RMS) of the PTB7 film is 0.32 nm (see Supplementary Fig. S7 online). Such a smooth interface is free from carrier trapping and is advantageous for the high branching ratio of the carrier escape.

## Summary

In summary, we investigated the interrelation between the instantaneous carrier density (*n*) per D/A interface area and the carrier density (*n*_collected_) collected as photocurrent in PTB7/C_70_ HJ device. By means of the time-resolved spectroscopy, we confirmed that the exciton-to-carrier conversion process takes place within ~1 ps at the D/A interface of the PTB7/C_70_ HJ device. We further determined the absolute magnitude of *n* by combination of the time-resolved and electrochemical spectroscopies. We found that the carrier recombination becomes dominant if *n* exceeds a critical concentration (*n*_c_ = 0.003 carriers/nm^2^). A phenomenological rate equation with carrier recombination term (−α*n*^3^) well reproduces the experimental relation between *n* and *n*_collected_. We confirmed that a similar behavior is observed in the PTB7/PC_71_BM BHJ devices even though *n*_c_ is much lower than the value of the HJ device. Our quantitative investigation based on the HJ device demonstrates that the fast carrier escape from the interface region is indispensable for high PCE, because the carrier accumulation nonlinearly accelerates the carrier recombination process.

## Method

### Fabrication and characterization of the OSCs

PTB7/C_70_ HJ solar cell was fabricated with a structure of indium tin oxide (ITO)/poly-(3,4-ethylenedioxythiophene) (PEDOT): poly-(styrenesulfonate) (PSS) (40 nm)/PTB7 (18 nm)/C_70_ (25 nm)/bathocuproine (BCP) (5 nm)/MgAg. The patterned ITO (conductivity: 10 Ω/sq) glass was pre-cleaned in an ultrasonic bath of acetone and ethanol and then treated in an ultraviolet-ozone chamber. A thin layer of PEDOT:PSS (40 nm) was spin-coated onto the ITO and dried at 110 °C for 10 min on a hot plate in air. A neat PTB7 film was spin-coated from an *o*-dichlorobenzene (*o-*DCB) solution. PTB7 was purchased from Sigma-Aldrich and used as received. The atomic force microscope (AFM) image of the PTB7 film is flat and consists of small grains less than 100 nm. Then, C_70_ (25 nm) was deposited by vacuum evaporation. Finally, BCP and MgAg were deposited onto the active layer by conventional thermal evaporation at a chamber pressure lower than 5 × 10^−4^ Pa, which provided the devices with an active area of 2 × 5 mm^2^.

PTB7/PC_71_BM BHJ solar cell was fabricated with a structure of ITO/PEDOT:PSS (40 nm)/active layer (140 nm)/LiF (1 nm)/Al. The patterned ITO glass was pre-cleaned in an ultrasonic bath of acetone and ethanol and then treated in an ultraviolet-ozone chamber. A thin layer of PEDOT:PSS (40 nm) was spin-coated onto the ITO and dried in air at 110 °C for 10 min on a hot plate. The substrate was then transferred to an N_2_ glove box and dried again at 110 °C for 10 min on a hot plate. An *o-*DCB/1.8-diiodooctane (DIO) solution of PTB7 : PC_71_BM with a ratio of 2 : 3 by weight (8 : 12 mg/mL) was subsequently spin-coated onto the PEDOT:PSS surface to form an active layer. Finally, LiF (1 nm) and Al (80 nm) were deposited onto the active layer by conventional thermal evaporation at a chamber pressure lower than 5 × 10^−4^ Pa. The active area of the OSCs is 2 × 5 mm^2^.

The *J* − *V* curves were measured using a voltage—current source/monitor under AM 1.5 solar-simulated light irradiation of 100 mW/cm^2^ (Bunkou-keiki, OTENTO-SUN III). The IPCE spectra was measured using a SM-250 system (Bunkou-keiki). The magnitudes of Φ_IQ_ at 400 nm were evaluated from the IPCE spectra with considering the absorption indexes: 0.61 and 0.84 for the HJ and BHJ devices, respectively.

### Pulse energy dependence of Φ_IQ_ of the OSCs

The pulse energy dependences of Φ_IQ_ of the OSCs were measured in a N_2_-filled box. For this experiment, the active area of the OSCs is 2 × 3 mm^2^. The excitation light pulse at 400 nm was generated as the second harmonics of a regenerative amplified Ti: sapphire laser in a β-BaB_2_O_4_ (BBO) crystal. The pulse width and repetition rate were 100 fs and 500 Hz, respectively. The maximum excitation intensity was 27 μJ/cm^2^. The photocurrents (*I*) from the OCSs were measured against the excitation pulse energy (*I*_photon_) per unit area of the device. The magnitudes of Φ_IQ_ were evaluated by *I* and *I*_photon_, with considering the absorption indexes: 0.61 and 0.84 for the HJ and BHJ devices, respectively.

### Femtosecond time-resolved spectroscopy

The time-resolved spectroscopy was performed in a pump-probe configuration. In order to reduce the irradiation damage, the blend films were placed in N_2_ atmosphere. The pump pulse at 400 nm was generated as the second harmonics of a regenerative amplified Ti: sapphire laser in a β-BaB_2_O_4_ (BBO) crystal. The pulse width, repetition rate, and pulse energy were 100 fs, 1000 Hz, and 27 μJ/cm^2^ respectively. The frequency of the pump pulse was decreased by half (500 Hz) to provide “pump-on” and “pump-off” conditions. A white probe pulse, generated by self-phase modulation in a sapphire plate was focused on the sample with the pump pulse. The spot sizes of the pump and probe pulses were 4.0 and 2.1 mm in diameter, respectively. The differential absorption (ΔOD) spectrum is expressed as -log*(I*_on_/*I*_off_) , where *I*_on_ and *I*_off_ are the transmission spectra under the pump-on and pump-off conditions, respectively.

Films prepared on quartz substrates were used in the time-resolved spectroscopies. PTB7/C_70_ bilayer film was prepared as follows. First, PTB7 neat film (18 nm) was spin-coated on quartz substrate from an *o-*DCB solution. The spin-coated film was dried in an inert N_2_ atmosphere. Then, C_70_ (25 nm) was deposited by vacuum evaporation. PTB7/PC_71_BM blend film was spin-coated on quartz substrate from a mixed solvent of *o-*DCB /DIO (97.5 : 2.5 vol %) of PTB7 : PC_71_BM with a ratio of 2 : 3 by weight (8 : 12 mg/mL). PTB7 neat film was spin-coated on quartz substrate from *o-*DCB solution. The spin-coated films were dried in an inert N_2_ atmosphere. The thicknesses of the PTB7 and PTB7/PC_71_BM blend films were 100 and 140 nm, respectively.

### Pulse energy dependence of Φ_CF_

The magnitude of Φ_CF_ (=α_photon_/α_D+_) can be calculated from the two quantities. α_D+_ (α_photon_) is the spectral intensity due to D^+^ per unit density of D^+^ (absorbed photons). α_D+_ is reported to be 0.013 nm^2^/carriers for PTB7^13^. In the PTB7-based OSCs, the carrier formation time is less than 1 ps. Therefore, we evaluated the α_D+_ values from the ΔOD spectra at 10 ps of the PTB7/C_70_ bilayer and PTB7/PC_71_BM blend films with considering the absorption indexes: 0.37 and 0.60 for the bilayer and blend films, respectively.

## Additional Information

**How to cite this article**: Moritomo, Y. *et al.* Carrier density effect on recombination in PTB7-based solar cell. *Sci. Rep.* **5**, 13648; doi: 10.1038/srep13648 (2015).

## Supplementary Material

Supplementary Information
