# Supplementary material for: Carrier density effect on recombination in PTB7-based solar cell
Source: Sci Rep. 2015 Sep 1;5:13648. doi: 10.1038/srep13648 (PMC4555099; doi:10.1038/srep13648)
Supplement: Supplementary Information [file srep13648-s1.pdf]

Supporting materials

Carrier density effect on recombination in PTB7-based solar cell

Yutaka Moritomo[1,2], Kouhei Yonezawa[1] and Takeshi Yasuda[3]

[1]Graduate School of Pure and Applied Science, Univ. of Tsukuba, Tsukuba 305-8571, Japan

[2]Center for Integrated Research in Fundamental Science and Engineering (CiRfSE), Univ. of Tsukuba, Tsukuba 305-8571, Japan

[3]Photovoltaic Materials Unit, National Institute for Materials Science (NIMS), Tsukuba, Ibaraki 305-0047, Japan

Correspoding Author: Yutaka Moritomo

e-mail: moritomo.yutaka.gf@u.tsukuba.ac.jp

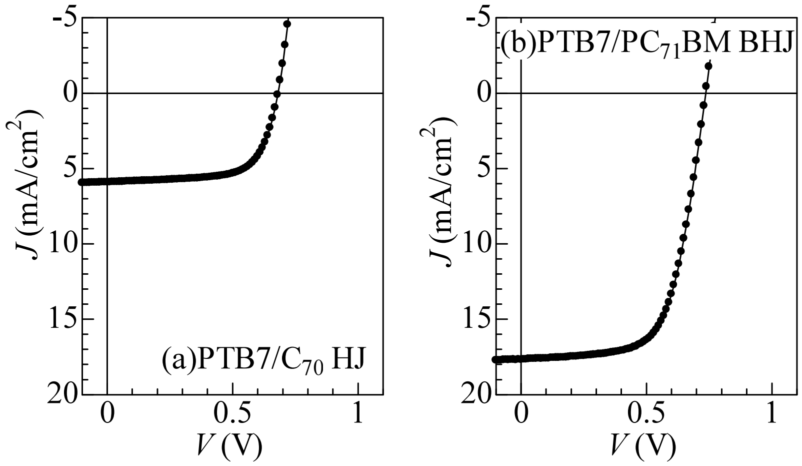

Fig. S1: $J-V$ curves of (a) PTB7/C_{70} HJ and (b) PTB7/PC_{71}BM BHJ solar cells under AM 1.5 solar-simulated light irradiation of 100 mW/cm^2.

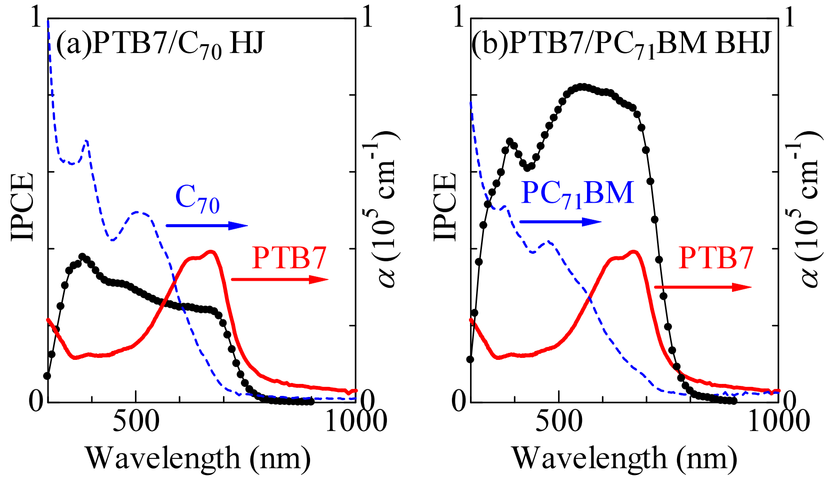

Fig S2: IPCE spectrum (a) PTB7/C_{70} HJ and (b) PTB7/PC_{71}BM BHJ solar cells together with absorption spectra of PTB7, C_{70}, and PC_{71}BM neat films. The configuration of HJ and BHJ solar cells are ITO/PEDOT:PSS (40 nm)/PTB7 (18 nm)/C_{70} (25 nm)/BCP (5 nm)/MgAg and ITO/PEDOT:PSS (40 nm)/BHJ (89 nm)/LiF (1 nm)/Al, respectively.

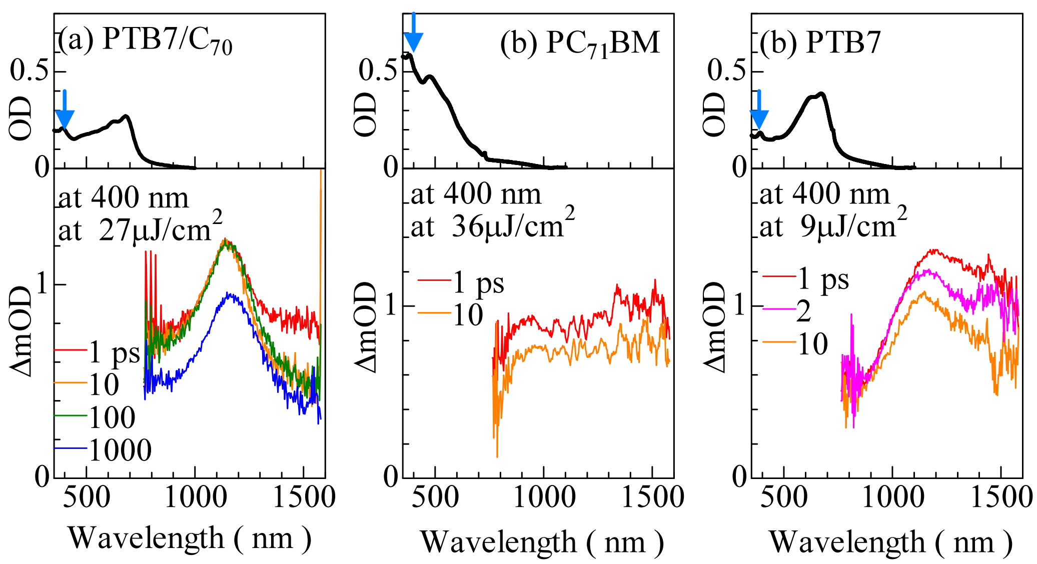

Fig. S3: Absorption (OD) spectra and differential absorption (ΔOD) spectra of (a) $PTB7/C_{70}$ bilayer, (b) $PC_{71}BM$ neat, and (c) PTB7 neat films at 300 K. The OD and ΔOD spectra in (b) and (c) were replotted from ref.10. Downward arrows indicated the excitation wavelength.

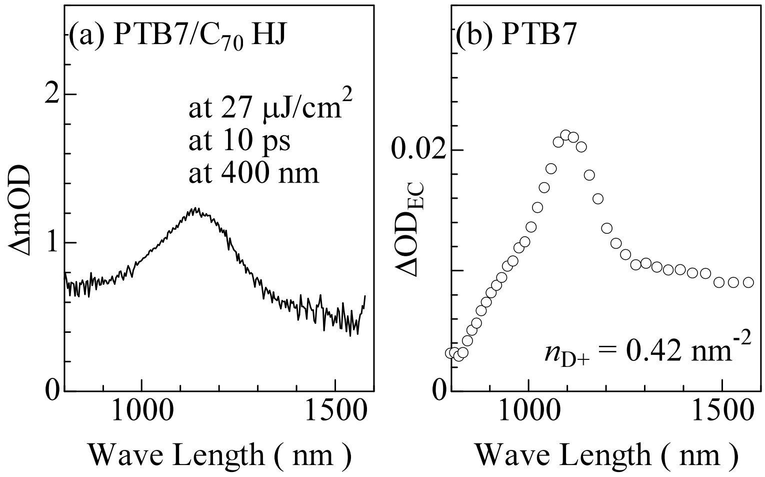

Fig. S4: (a) ΔOD spectrum of the $PTB7/C_{70}$ bilayer film at 10 ps. (b) electrochemical (ΔOD_{EC}) spectrum of the neat PTB7 film. The ΔOD_{EC} spectra in (b) were replotted from ref.13. The electrochemical differential absorption (ΔOD_{EC}) spectrum is expressed as $-\log(I_{doped}/I_{non})$, where I_{doped} and I_{non} are the transmission spectra of the electrochemically hole-doped and non-doped films, respectively. The carrier density (n_{D+}) per unit area of the device was calculated from the current density and doping time.

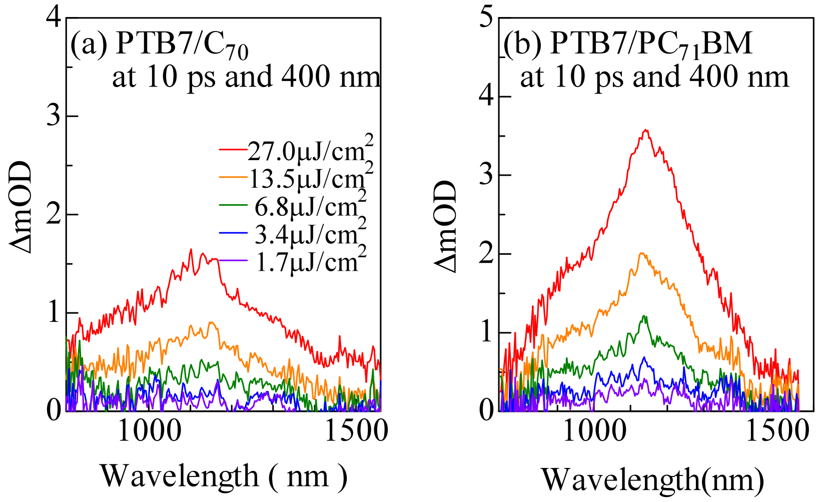

Fig. S5: Differential absorption (ΔOD) spectra at 10 ps in (a) PTB7/C_{70} bilayer and (b) PTB7/$PC_{71}BM$ blend films against excitation pulse energy.

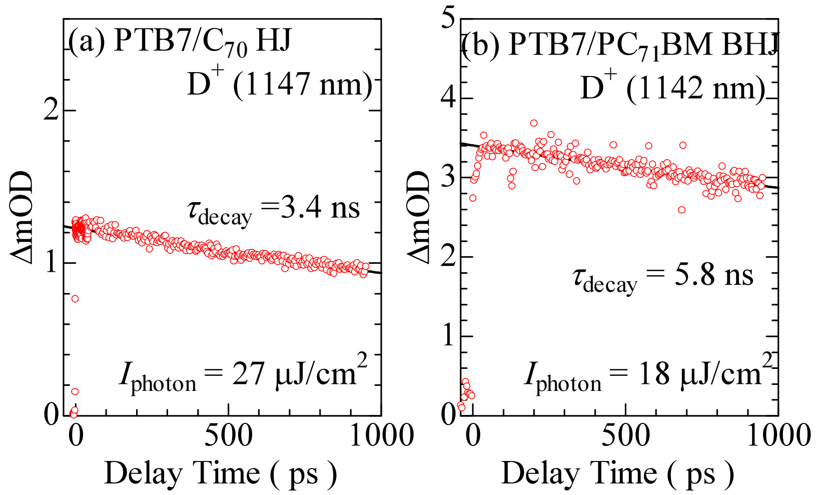

Fig. S6: Temporal evolutions of PIA signals due to D^+ in (a) PTB7/C_{70} bilayer film and (b) PTB7/$PC_{71}BM$ blend films. The data in (b) were replootted from ref.10. The excitation wavelength was 400 nm. The solid curves show the results of the least-squares fittings using an exponential function: $\Delta OD = A\exp(-t/\tau)$.

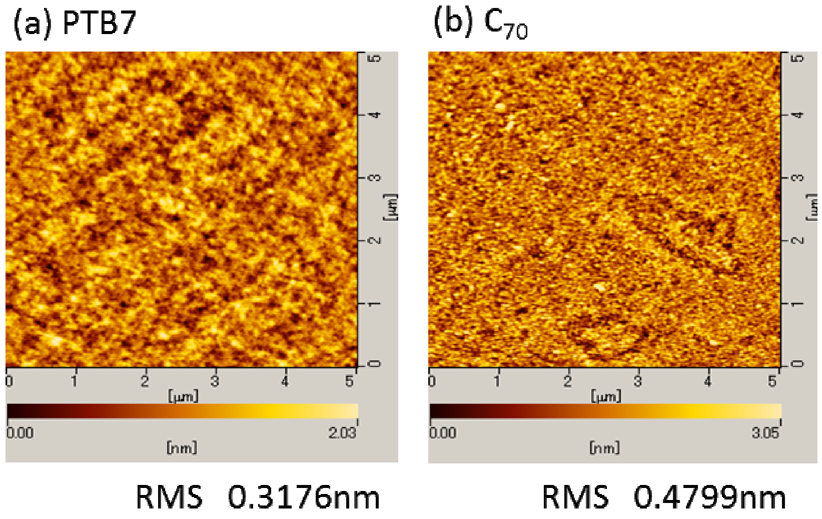

Fig. S7: Atomic force microscopy (AFM) images of (a) spin-coated PTB7 and (b) vacuum-evaporated C_{70} neat films. Root-mean-square (RMS) of roughness are 0.3176 nm and 0.4799 nm for the PTB7 and C_{70} neat films, respectively.
